# Supplementary material for: Mitochondria preserve an autarkic one-carbon cycle to confer growth-independent cancer cell migration and metastasis
Source: Nat Commun. 2022 May 16;13:2699. doi: 10.1038/s41467-022-30363-y (PMC9110368; doi:10.1038/s41467-022-30363-y)
Supplement: Supplementary file 1 — Supplementary Information [file 41467_2022_30363_MOESM1_ESM.pdf]

SUPPLEMENTARY INFORMATION Kiweler et al.

Mitochondria preserve an autarkic one-carbon cycle to confer growth-independent cancer cell migration and metastasis

Nicole Kiweler[1], Catherine Delbrouck[1,2], Vitaly I. Pozdeev[3], Laura Neises[1], Leticia Soriano-Baguet[2,4,5], Kim Eiden[1,2], Feng Xian[6], Mohaned Benzarti[1,2], Lara Haase[1,2,7], Eric Koncina[3], Maryse Schmoetten[3], Christian Jaeger[7], Muhammad Zaeem Noman[8], Alexei Vazquez[9], Bassam Janji[8], Gunnar Dittmar[2,6], Dirk Brenner[4,5,10], Elisabeth Letellier[3], Johannes Meiser[1]*

[1] Cancer Metabolism Group, Department of Cancer Research, Luxembourg Institute of Health, Luxembourg, Luxembourg;

[2] Faculty of Science, Technology and Medicine, University of Luxembourg, 2 avenue de Université, Esch-sur-Alzette, Luxembourg;

[3] Faculty of Science, Technology and Medicine, Department of Life Sciences and Medicine, Molecular Disease Mechanisms Group, University of Luxembourg, Esch-sur-Alzette, Luxembourg;

[4] Experimental & Molecular Immunology, Department of Infection and Immunity, Luxembourg Institute of Health, 29 Rue Henri Koch, Esch-sur-Alzette, Luxembourg

[5] Immunology & Genetics, Luxembourg Centre for Systems Biomedicine, University of Luxembourg, 7 Avenue des Hauts Fourneaux, Esch-sur-Alzette, Luxembourg

[6] Proteomics of cellular signaling, Department of Infection and Immunity, Luxembourg Institute of Health,1a Rue Thomas Edison, Strassen, Luxembourg

[7] Luxembourg Centre for Systems Biomedicine, University of Luxembourg, Esch-sur-Alzette, Luxembourg;

[8] Tumor Immunotherapy and Microenvironment (TIME) Group, Department of Cancer Research, Luxembourg Institute of Health, Luxembourg, Luxembourg

[9] Institute of Cancer Sciences, University of Glasgow, Glasgow, United Kingdom

[10] Odense Research Center for Anaphylaxis (ORCA), Department of Dermatology and Allergy Center, Odense University Hospital, University of Southern Denmark, Odense, Denmark;

*Correspondence:

Email: johannes.meiser@lih.lu

Twitter: @JohannesMeiser

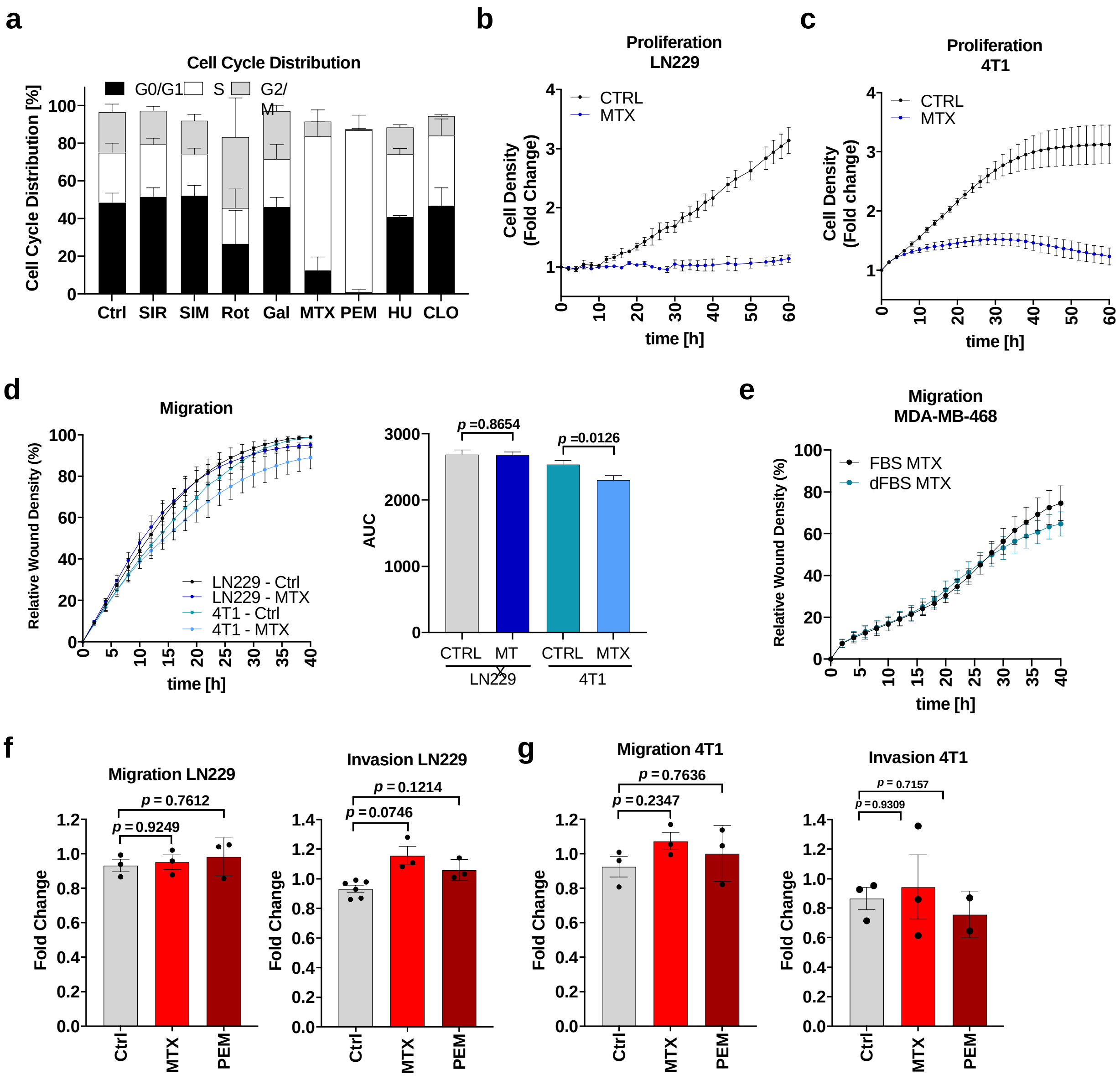

Supplementary Figure 1: (A) MDA-MB-468 cells were treated for 48 h with 100 nM Sirolimus (SIR), 1 µM Simvastatin (SIM), 50 nM Rotenone (Rot), galactose (Gal) supplementation, 50 nM Methotrexate (MTX), 1 µM Pemetrexed (PEM), 0.5 mM hydroxyurea (HU), and 100 nM Clofarabine (CLO). Cells were analyzed for cell cycle distribution by flow cytometry of PI-stained cells; mean ± SD of independent experiments (n = 11 for CTRL; n = 4 for SIR, MTX, PEM; n = 3 for SIM, Rot, Gal, HU, CLO). (B) Proliferation of LN229 cells in response to 50 nM MTX; mean ± SEM of independent experiments (n = 3). (C) Proliferation of 4T1 cells in response to 75 nM MTX; mean ± SEM of independent experiments (n = 4). (D) Migration of LN229 and 4T1 cells in response to 50 (LN229) or 75 (4T1) nM MTX and respective AUC; mean ± SEM of independent experiments (n = 5); unpaired, two-tailed t-test with Welch's correction. (E) Migration of MDA-MB-468 cells after 24 h treatment with 50 nM MTX in medium supplemented with normal or dialyzed FBS; graph shows mean ± SEM of four independent experiments. (F), (G) Migration and invasion of LN229 (F) and 4T1 (G) cells after 24h treatment with 50 nM (F), 75 nM (G) MTX was assessed using ECM-Collagen-coated or non-coated Boyden chambers. Each dot represents an independent experiment; mean ± SEM (n = 3); Brown-Forsythe and Welch one-way ANOVA with Dunnett's multiple comparisons test. Source data are provided as a Source Data file.

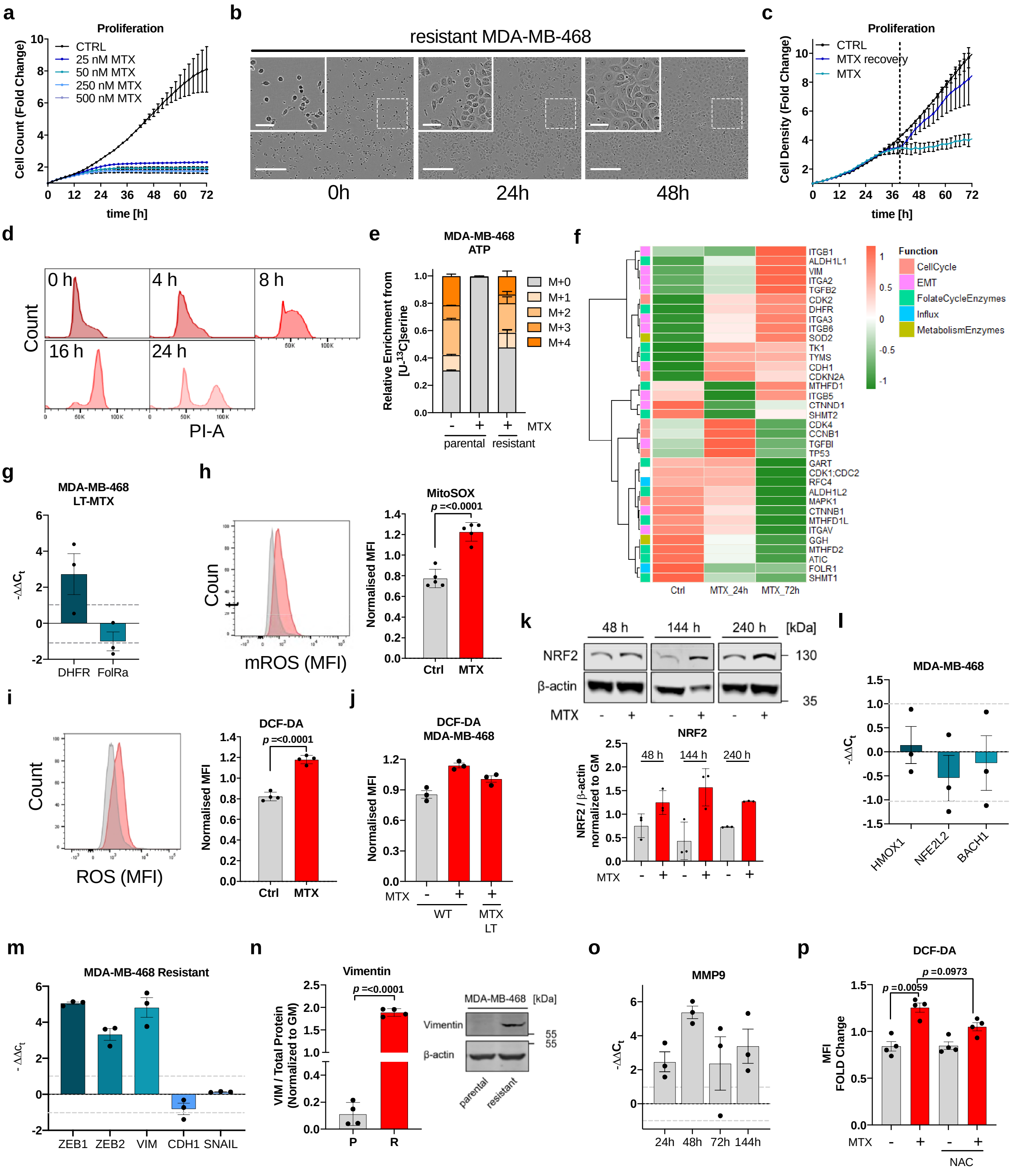

Supplementary Figure 2: (A) Proliferation of NucLight Rapid Red stained MDA-MB-468 cells upon the indicated concentrations of MTX; mean ± SEM of independent experiments (n = 3). **(B)** Morphology of proliferating MTX-resistant MDA-MB-468 cells. Bright-field images are representative of independent experiments. Scale bars correspond to 60 and 300 µm. **(C)** Growth of MDA-MB-468 cells in response to 50 nM MTX and growth recovery upon removal of MTX after 38 h assessed as fold change of cell density; mean ± SEM of independent experiments (n = 3). **(D)** Recovery of cell cycle profile at the indicated time points after MTX removal as in (C). Histograms are representative of three independent experiments. **(E)** MID of intracellular ATP upon [U-^{13}C]serine tracer in response to 24 h 50 nM MTX in parental MDA-MB-468 cells and MTX-resistant MDA-MB-468 cells. Graph shows mean ± SEM of two independent experiments each measured in triplicate wells. **(F)** Heatmap of selected protein expressions from SILAC quantitative proteomics measurement with different MTX

treatment durations. The log2 fold changes of each protein were standardized by row. Molecular function of proteins are indicated with colors. **(G)** mRNA expression from the indicated genes was quantified by real-time RT-qPCR in resistant long-term (LT) MTX treated MDA-MB-468 cells relative to MDA-MB-468 WT cells. Each dot represents one independent experiment; mean ± SD (n = 3). (**H**) Mitochondrial ROS levels in response to 24 h 50 nM MTX measured by flow cytometric quantification of MitoSOX mean fluorescence intensity. Each dot represents an independent experiment; mean ± SD (n = 5); unpaired, two-tailed t-test with Welch's correction. **(I)** Intracellular ROS levels in response to 24 h 50 nM MTX measured by flow cytometric quantification of DCF-DA mean fluorescence intensity. Each dot represents an independent experiment; mean ± SD (n = 4); unpaired, two-tailed t-test with Welch's correction. **(J)** Intracellular ROS levels in response to 24 h MTX treatment in MDA-MB-468 WT cells and in LT MTX treated MTX_resistant MDA-MB-468 cells measured by flow cytometric quantification of DCF-DA mean fluorescence intensity. Each dot represents one independent experiment; mean ± SD (n = 3). (**K**) NRF2 expression in MDA-MB-468 cells after treatment with 50 nM MTX for the indicated time was quantified by Western Blot. Normalized signal intensity was quantified relative to β-actin signal. Each dot represents an independent experiment; mean ± SD (n = 3). (**L**) mRNA expression from the indicated target genes in MDA-MB-468 cells was measured after 144 h 50 nM MTX and quantified relative to untreated cells using real-time RT-qPCR. Each dot represents one independent experiment; mean ± SD (n = 3). (**M**) mRNA expression from the indicated target genes in MTX-resistant MDA-MB-468 cells relative to parental MDA-MB-468 cells as measured using real-time RT-qPCR. Each dot represents an independent experiment; mean ± SEM (n = 3). **(N)** Expression of vimentin in parental and MTX-resistant MDA-MB-468 cells; β-actin serves as loading control. Quantification of vimentin signal intensity relative to total protein stain. Each dot represents an independent experiment; mean ± SD (n = 4); unpaired, two-tailed t-test with Welch's correction. (**O**) mRNA expression of MMP9 was quantified in MDA-MB-468 cells after 50 nM MTX at the indicated time points using real-time RT-qPCR. Each dot represents an independent experiment; mean ± SEM (n = 3). **(P)** Intracellular ROS levels in MDA-MB-468 cells in response to 24 h 50 nM MTX and 10 mM NAC as measured by flow cytometric quantification of DCF-DA mean fluorescence intensity. Each dot represents an independent experiment. Graph shows mean ± SD (n = 4); Brown-Forsythe and Welch ANOVA test with Games-Howell's multiple comparisons test. Source data are provided as a Source Data file.

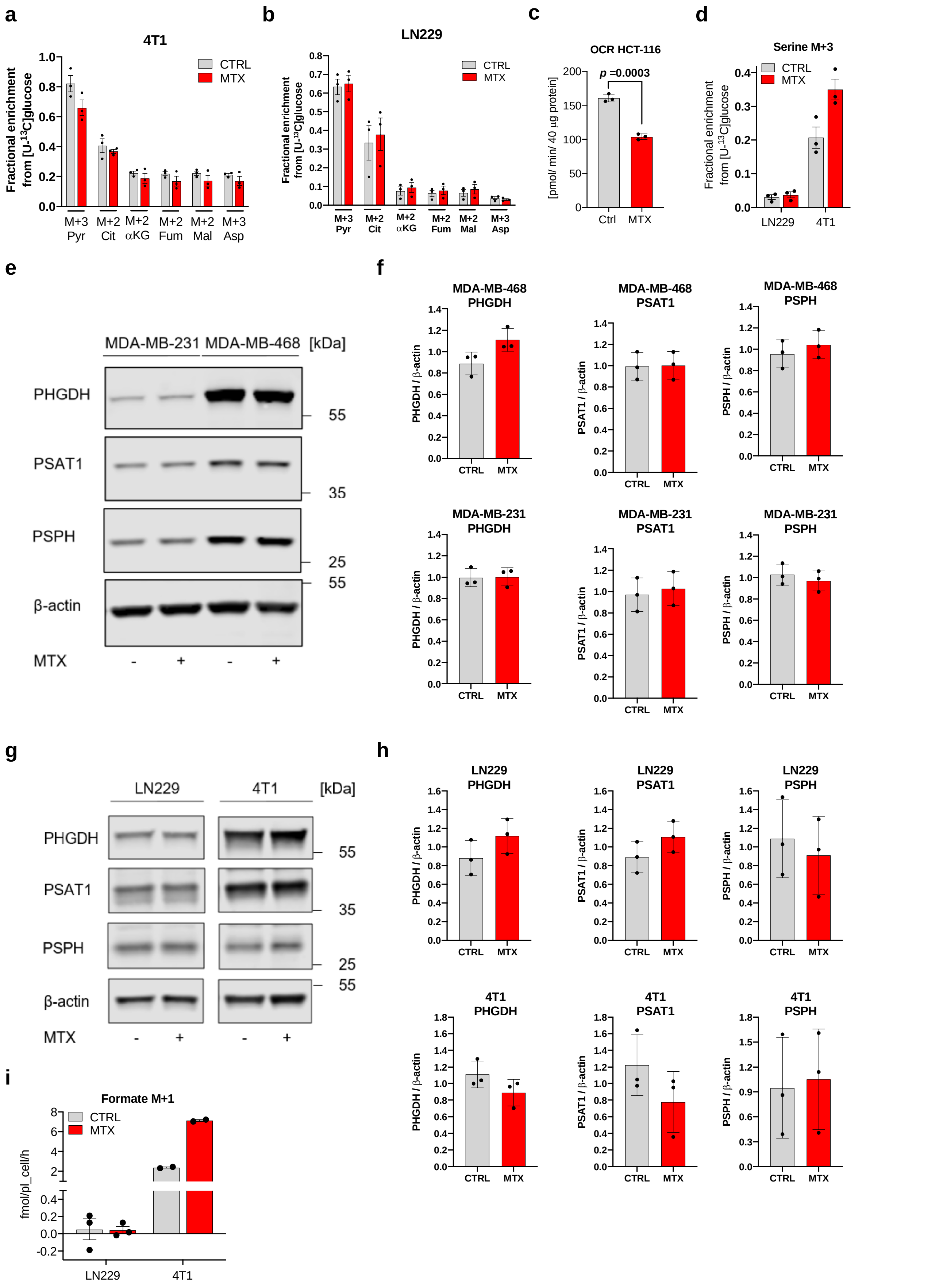

Supplementary Figure 3: (A), (B) Relative isotopologue abundance following [U-^{13}C]glucose tracing in 4T1 (A) and LN229 (B) cells in response to 24 h 75 nM MTX (A) and 50 nM MTX (B). Each dot represents an individual experiment composed

of triplicate wells; mean ± SEM (n = 3). **(C)** Basal cellular respiration in response to 24 h 50 nM MTX treatment was determined in HCT-116 cells as quantification of mitochondrial OCR. Each dot represents an individual experiment composed of six technical replicates; mean ± SEM (n = 3); unpaired, two-tailed t-test with Welch's correction. **(D)** M+3 isotopologue abundance of serine upon [U-^{13}C]glucose tracing in response to 24 h 50 nM MTX in LN229 and 24 h 75 nM MTX in 4T1 cells. Each dot represents an independent experiment in triplicate wells; mean ± SEM (n = 3). **(E)** Expression of PHGDH, PSAT1, and PSPH in MDA-MB231 and MDA-MB-468 cells upon 24 h 50 nM MTX treatment; β-actin serves as loading control. **(F)** Treatment as in (E). Quantification of PHGDH, PSAT1, and PSPH Western blot signal intensity relative to β-actin normalized to global mean in MDA-MB231 and MDA-MB-468 cells. Each dot represents an independent experiment; mean ± SD (n = 3). **(G)** Expression of PHGDH, PSAT1, and PSPH in LN229 and 4T1 cells upon 24 h 50 nM MTX (LN229) and 75 nM MTX (4T1) treatment; β-actin serves as loading control. **(H)** Treatment as in (G). Quantification of PHGDH, PSAT1, and PSPH Western blot signal intensity relative to β-actin and normalized to global mean in LN229 and 4T1 cells. Each dot represents an independent experiment; mean ± SD (n = 3). **(I)** M+1 formate release rate in LN229 and 4T1 cells after 24 h [U-^{13}C]glucose tracing and treatment with 50 nM (LN229) and 75 nM (4T1) MTX. Each dot indicates an independent experiment measured in triplicate wells; mean ± SEM (n = 3 (LN229), n = 2 (4T1)). Source data are provided as a Source Data file.

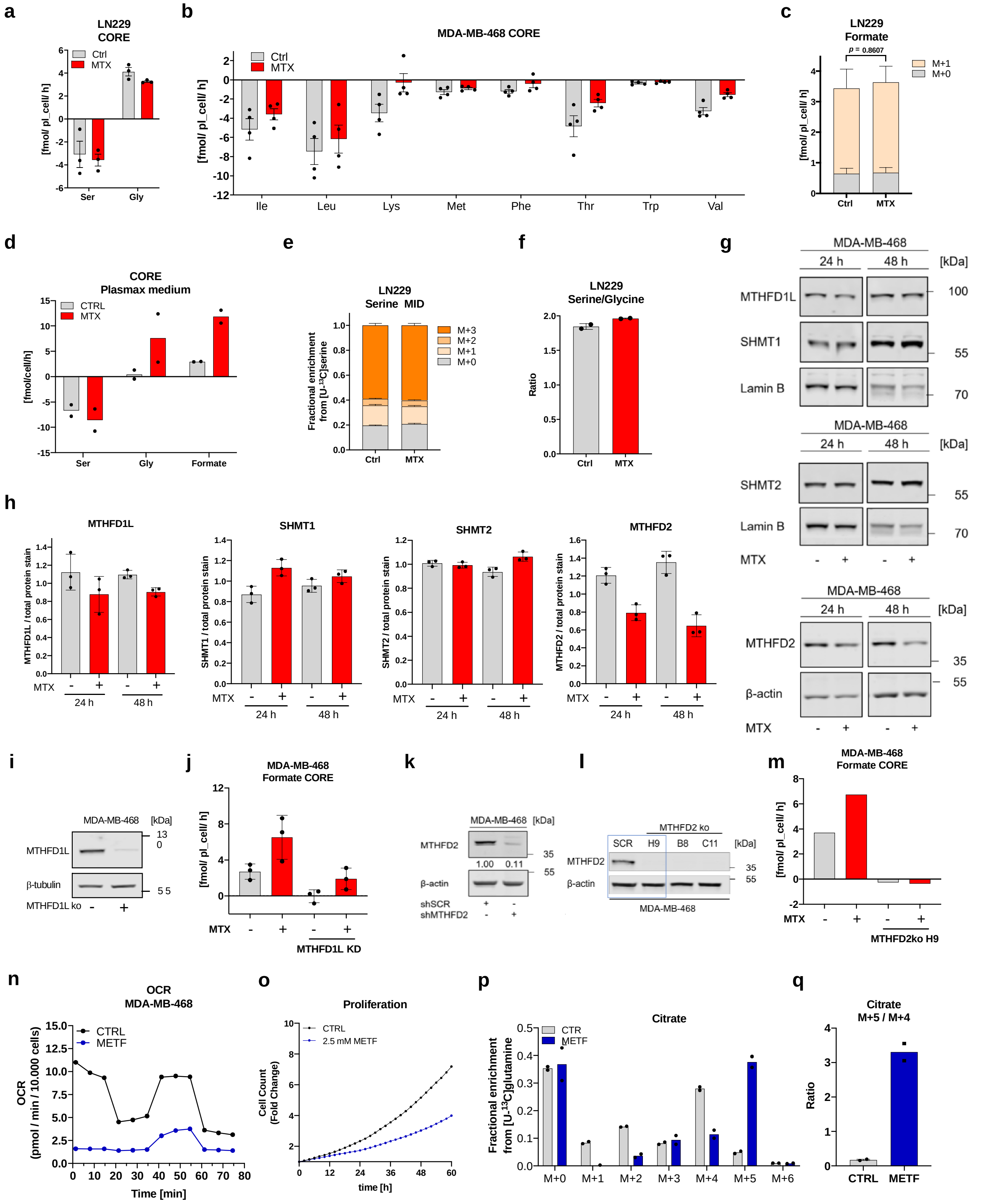

Supplementary Figure 4: (A) Absolute CORE rates of serine and glycine of LN229 cells in response to 24 h 50 nM MTX. Each dot represents the mean of an individual experiment each measured in triplicate wells; mean ± SEM. **(B)** Absolute CORE rates of essential amino acids as indicated in response to 24 h 50 nM MTX. Each dot represents an independent experiment in triplicate wells; mean ± SEM (n = 3). **(C)** Formate release rates of M+0 and M+1 formate isotopologues upon [U-^{13}C]serine tracing in LN229 cells in response to 24 h 50 nM MTX. Graph shows mean ± SEM of three independent experiments each measured in triplicate wells; unpaired, two-tailed t-test with Welch's correction. **(D)** Absolute CORE rates of serine, glycine, and formate in MDA-MB-468 cells in response to 24 h 50 nM MTX in Plasmax medium. Mean of two independent experiments in triplicate wells. **(E)** MID of intracellular serine upon [U-^{13}C]serine tracing in LN229 cells in response to 24 h 50 nM MTX; mean ± SEM of two independent experiments each measured in triplicate wells. **(F)** Ratio

of intracellular serine and glycine levels in response to 24 h 50 nM MTX in LN229 cells. Mean ± SEM of independent experiments in triplicate wells (n = 2). **(G)** Expression of MTHFD1L, SHMT1, SHMT2, and MTHFD2 in MDA-MB-468 cells 24 h and 48 h 50 nM MTX; β-actin serves as loading control. **(H)** Treatment as in (G). Quantification of MTHFD1L, SHMT1, SHMT2, and MTHFD2 Western blot signal intensity relative to total protein stain normalized to GM in MDA-MB-468 cells. Each dot represents an independent experiment; mean ± SD (n = 3). **(I)** Residual MTHFD1L protein expression in MDA-MB-468 MTHFD1L CRISPR KO cells was determined on Western Blot. β-tubulin serves as loading control. **(J)** Absolute CORE rates of formate from mock or shMTHFD1L-transfected MDA-MB-468 cells upon 24 h 50 nM MTX. Dots represent technical replicates of a representative experiment; mean ± SD (n = 3). **(K)** Residual MTHFD2 protein expression in mock or shMTHFD2-transfected MDA-MB-468 cells was determined on Western Blot. β-actin serves as loading control. **(L)** Residual MTHFD2 protein expression in three MDA-MB-468 MTHFD2 CRISPR KO clones was determined on Western Blot. β-actin serves as loading control. **(M)** Absolute CORE rates of formate from MDA-MB-468 cells upon depletion of MTHFD2 (CRISPR clone H9) and 24 h 50 nM MTX. Plot shows the median of one experiment done in triplicate wells. **(N)** Oxygen consumption rate (OCR) in MDA-MB-468 cells after 24h treatment with 2.5 mM Metformin. Graph shows mean of six technical replicates. **(O)** Proliferation of MDA-MB-468 cells in response to treatment with 2.5 mM Metformin. Graph shows mean of two independent experiments. **(P)** Citrate MID from [U-^{13}C]glutamine tracing in MDA-MB-468 cells after 24 h treatment with 2.5 mM Metformin. Graph shows mean of two independent experiments. Dots indicate the mean of each independent experiment performed in triplicate wells. **(Q)** Ratio of M+5/M+4 istotopologues of citrate after 24 h treatment with 2.5 mM Metformin and tracing with [U-^{13}C]glutamine tracing. Graph shows mean of two independent experiments. Dots indicate the mean of each independent experiment performed in triplicate wells. Source data are provided as a Source Data file.

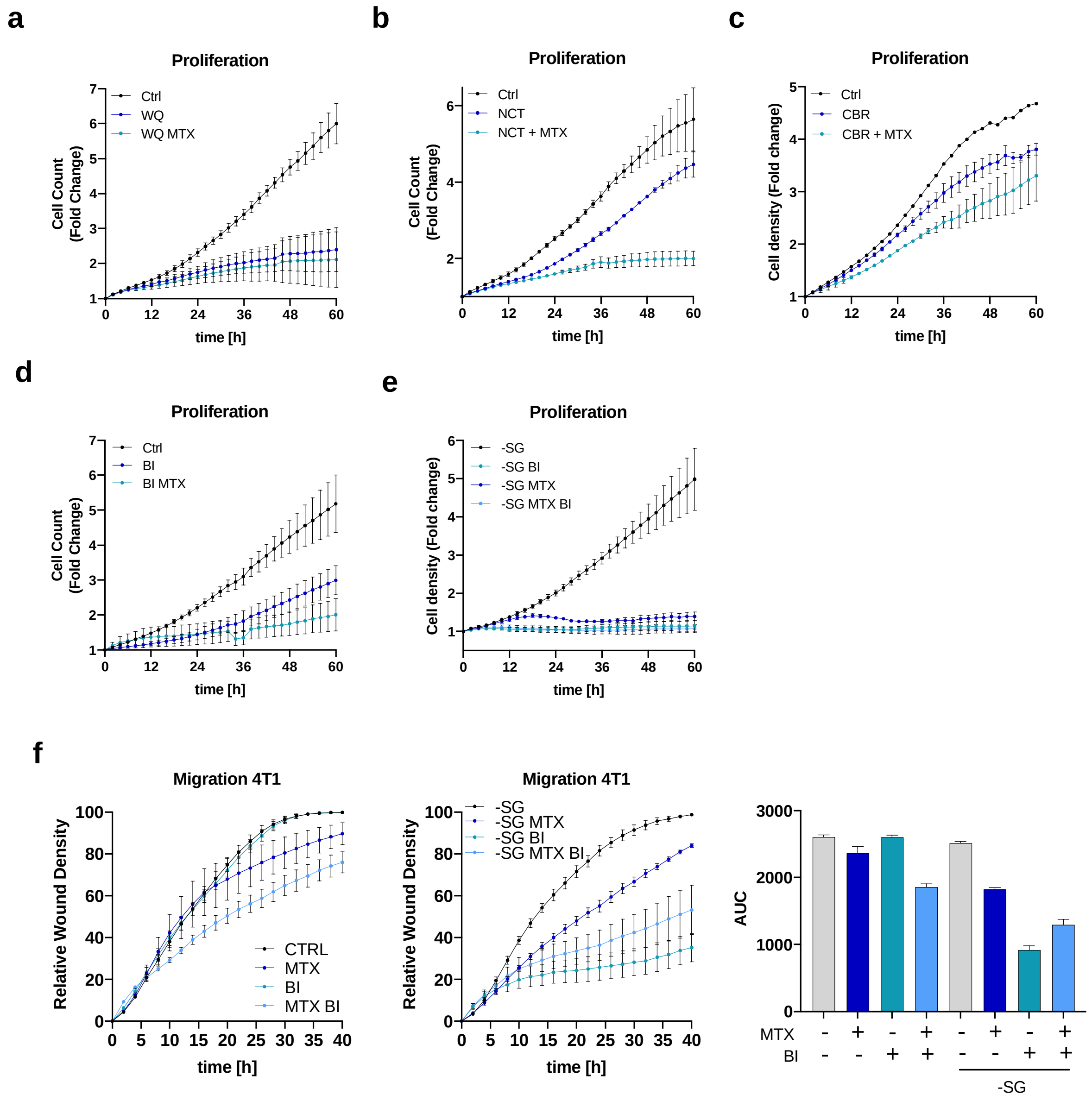

Supplementary Figure 5: (A) Proliferation of NucLight Rapid Red stained MDA-MB-468 cells upon treatment with 10 µM WQ-2101 and 50 nM MTX as indicated; mean ± SEM of independent experiments (n = 6). **(B)** Proliferation of NucLight Rapid Red stained MDA-MB-468 cells upon treatment with 10 µM NCT-502 and 50 nM MTX as indicated; mean ± SEM of independent experiments (n = 4). **(C)** Proliferation of MDA-MB-468 cells measured as fold cell density upon treatment with 30 µM CBR-5884 and 50 nM MTX as indicated; mean ± SEM of independent experiments (n = 1 (CTRL), n = 2 (CBR, CBR+MTX)). **(D)** Proliferation of NucLight Rapid Red stained MDA-MB-468 cells upon treatment with 15 µM BI-4916 and 50 nM MTX as indicated; mean ± SEM of independent experiments (n = 7). **(E)** Proliferation of serine- and glycine-starved MDA-MB-468 cells measured as fold cell density upon treatment with 15 µM BI-4916 and 50 nM MTX as indicated; mean ± SEM of independent experiments (n = 3). (**F**) Migration of 4T1 cells upon treatment with 75 nM MTX and 15 µM BI in the presence or absence of serine and glycine in culture medium and respective AUC over 40 h. Graph shows mean ± SD of one representative experiment performed in technical replicates (n = 3 for all –SG conditions, n = 4 for all others). Source data are provided as a Source Data file.

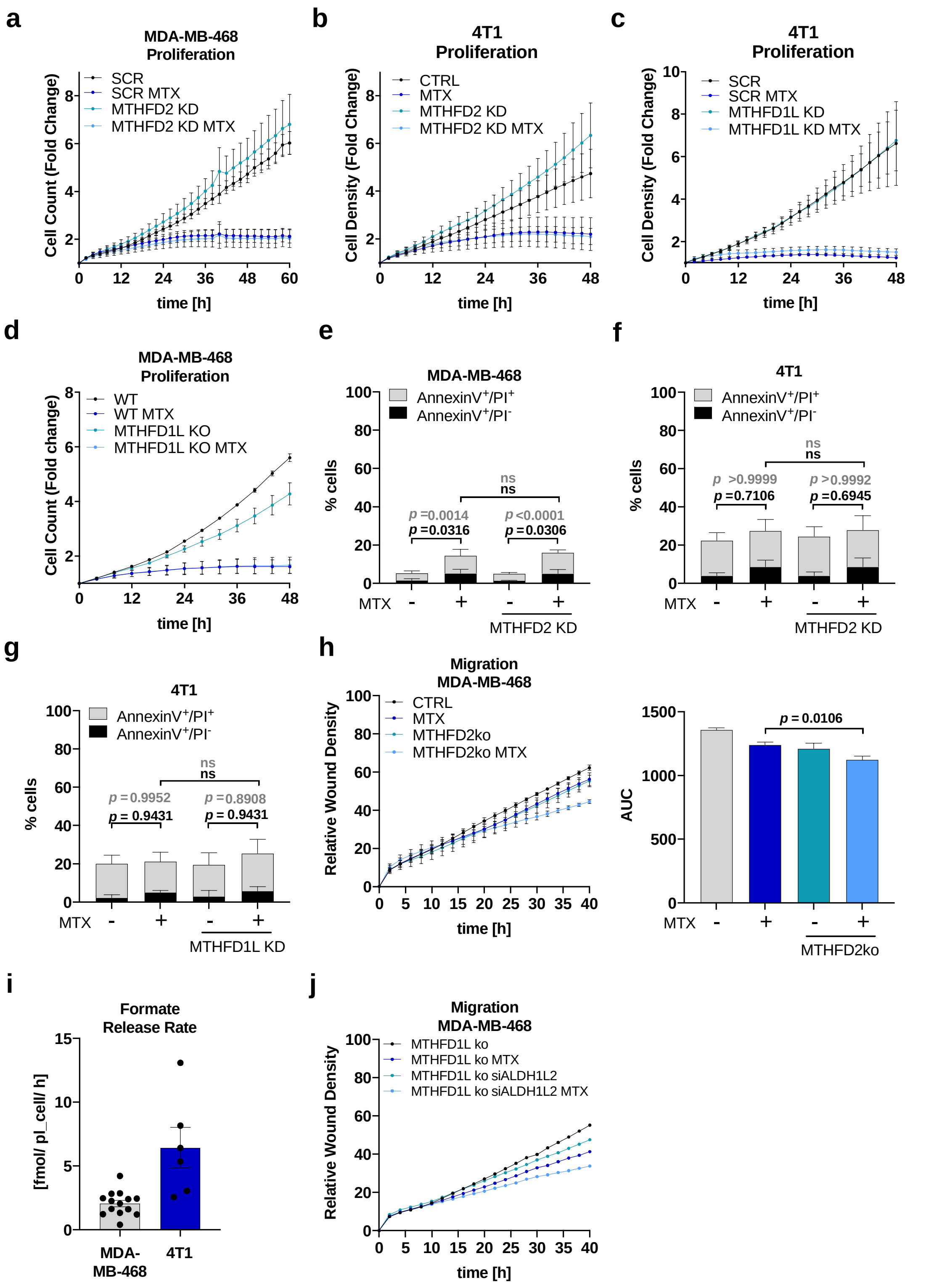

Supplementary Figure 6: (A-D) Proliferation of MDA-MB-468 (A,D) and 4T1 (B,C) cells upon MTHFD1L and MTHFD2 depletion as indicated after treatment with 50 nM MTX (A,D) or 75 nM MTX (B,C). Graph shows mean ± SEM of independent experiments (n = 6 (A); 3 (B); 1 (SCR MTX), 3 (SCR, MTHFD1LKD MTX) or 4 (MTHFD1LKD) (C); 4 (D)). (**E-G**) MDA-MB-468 (E) and 4T1 (F,G) cells depleted for MTHFD2 or MTHFD1L were treated with 50 nM (E) or 75 nM (F, G) MTX for 48h. Cell death was assessed by flow cytometry and AnnexinV-FITC/PI-staining; mean ± SD of independent experiments (n = 4). 2-way ANOVA with Dunnett's multiple comparisons test. **(H)** Migration of MDA-MB-468 cells (clone H9) in response to MTHFD2 knockout and 50 nM MTX treatment. Graph shows mean ± SEM (n = 3); Brown-Forsythe and Welch one-way ANOVA with Dunnett's multiple comparisons test. **(I)** Absolute formate release from MDA-MB-468 and 4T1

cells. Graph shows mean ±SEM of independent experiments, each performed in triplicate wells (n = 14 (MDA-MB-468), n = 6 (4T1)). **(J)** Migration of MDA-MB-468 MTHFD1L knockout cells in response to siRNA-mediated knockdown of ALDH1L2 and 50 nM MTX treatment. Graph shows mean of technical replicates (n = 7 (MTHFD1Lko, MTHFD1lko MTX) – 8 (MTHFD1Lko siALDH1L2, MTHFD1Lko siALDH1L2 MTX) wells). Source data are provided as a Source Data file.

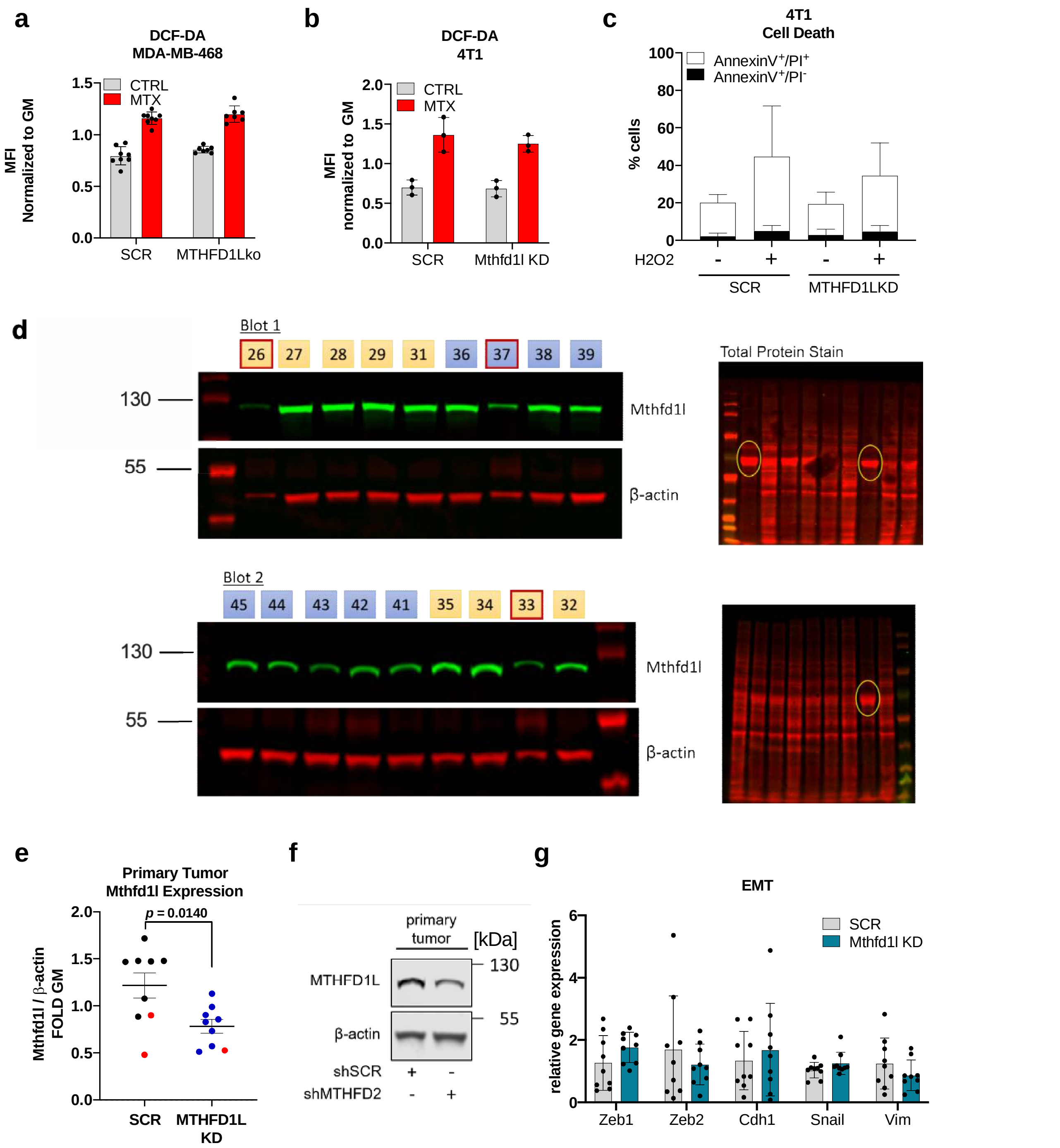

Supplementary Figure 7: (A, B) Intracellular ROS levels in MDA-MB-468 and 4T1 cells in response to 24 h 50 nM MTX (A) or 75 nM MTX (B) measured by flow cytometric quantification of DCF-DA mean fluorescence intensity. Each dot represents one independent experiment; mean ± SD (n = 7 (MTHFD1Lko) – 8 (SC) (A) or n = 3 (B)). **(C)** 4T1 cells depleted or not for Mthfd1l were treated with 500 µM H_2O_2 for 24 h. Cell death was assessed by flow cytometry and AnnexinV-FITC/PI-staining; mean ± SD of independent experiments (n = 4). **(D)** Western blot images of protein lysates from primary tumor tissue. Indicated replicates (red frame around box) were excluded from analysis in Figure 7D, due to the here presented finding that total protein in these samples was mainly composed of a single protein (see Total Stain). This indicates unreliability of the sample. (E) Mthfd1l expression in primary tumor tissue obtained by orthotopic implantation of 4T1 SCR and Mthfd1lKD breast cancer cells. Red dots indicate data points that were excluded as explained in (D); mean ± SD (n = 9) unpaired, two-tailed t-test with Welch's correction.**(F)** Representative Western Blot image of Mthfd1l protein expression in primary tumor tissue in mice after orthotopic implantation of 4T1 scramble or Mthfd1l shRNA transfected breast cancer cells. β-actin serves as loading control. (**G**) mRNA expression from indicated target genes in primary tumor

tissue in mice after orthotopic implantation of 4T1 scramble or Mthfd1l shRNA transfected breast cancer cells measured by real-time RT qPCR. Each dot indicates one individual animal; mean ± SD (n = 9). Source data are provided as a Source Data file.

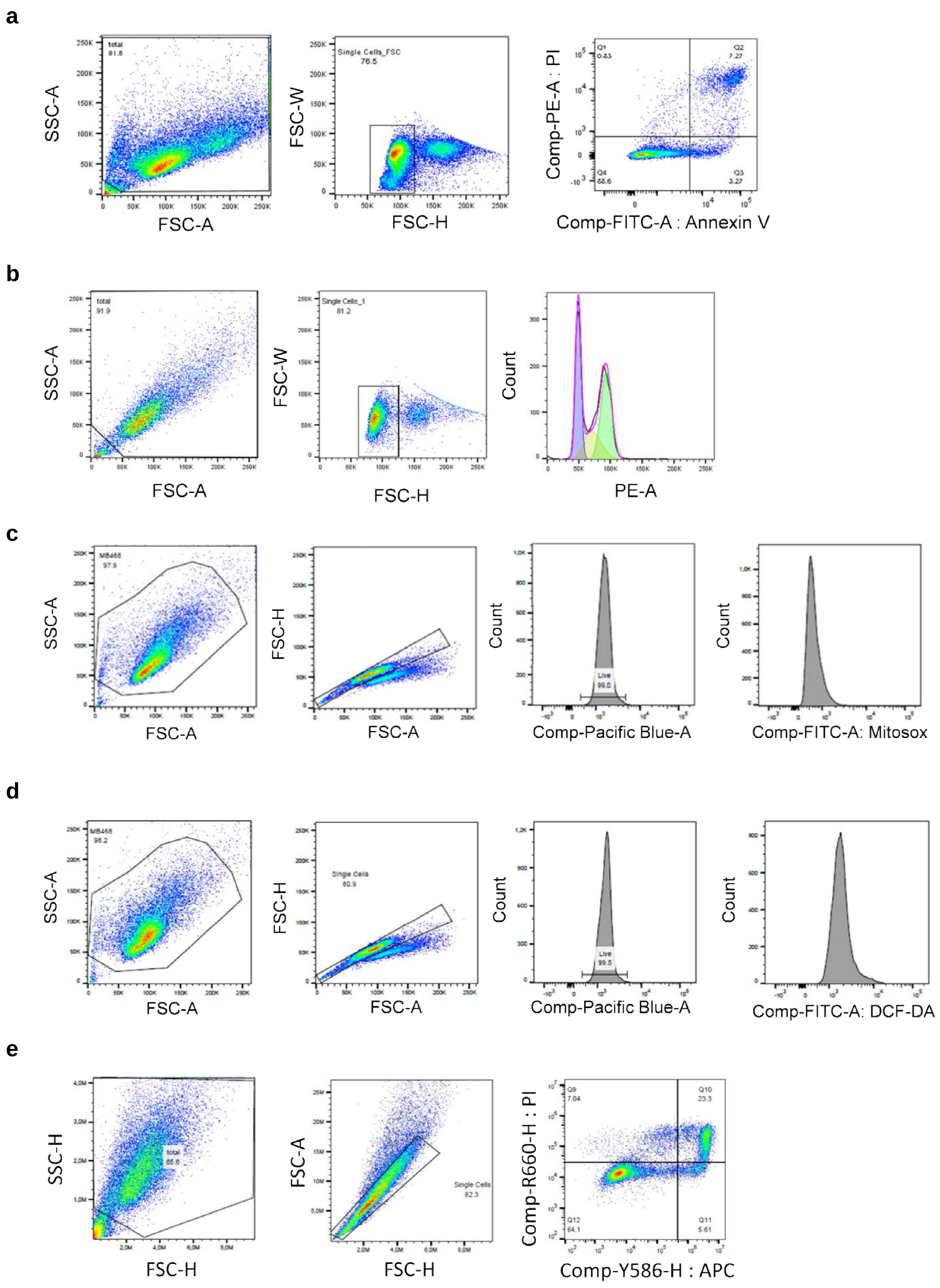

Supplementary Figure 8: (A) Exemplary gating for Annexin-PI measurement using Annexin-V-FITC and PI. **(B)** Exemplary gating for cell cycle analysis using automatic cell cycle analysis tool in FlowJo. **(C)** Exemplary gating for

mitochondrial ROS measurement using Mitosox dye. **(D)** Exemplary gating for cytosolic ROS measurement using DCF-DA dye. (**E**) Exemplary gating for Annexin-PI measurement using Annexin-V-APC and PI in GFP-positive cells.

Supplementary Table 1. Dwell times as well as quantification ions (m/z) for formate compounds.

Derivative (Metabolite)	Quant-Ion (m/z)	Dwell Time (ms)
Benzyl formate (Formic acid) – M_0	136.1	40
Benzyl formate (Formic acid) – M_1	137.1	40
IS Benzyl formate (Formic acid 13C, D) – M_0	138.1	40
IS Benzyl formate (Formic acid 13C, D) – M_1	139.1	40
IS Benzyl formate (Formic acid 13C, D) – M_2	140.1	40

Supplementary Table 2. Dwell times as well as quantification ions (m/z) for the measured compounds.

Derivative (m/z of selected fragment)	Quant-Ions (m/z)	Dwell Time (ms)	Sum Formula (Fragment)	MID size
Pyruvic Acid 1MeOX 1TBDMS (174)	174.0 – 180.1	15	C6H12O3NSi	4
Lactic Acid 2TBDMS (261)	261.1 – 267.1	15	C11H25O3Si2	4
Alanine 2TBDMS (260)	260.1 – 266.1	15	C11H26NO2Si2	4
Glycine 2TBDMS (246)	246.1 – 252.1	15	C10H24NO2Si2	3
Succinic Acid 2TBDMS (289)	289.1 – 296.1	15	C12H25O4Si2	5
Fumaric Acid 2TBDMS (287)	287.1 – 294.1	15	C12H23O4Si2	5
(Internal Standard) Pentanedioic-D6 Acid	235.2, 309.2, 351.3	30		
Serine 3TBDMS (390)	390.2 – 396.2	15	C17H40NO3Si3	4
a-Ketoglutaric Acid 1MeOX 2TBDMS (346)	346.2 – 354.2	10	C14H28NO5Si2	6
Malic Acid 2TBDMS (419)	419.2 – 426.2	15	C18H39O5Si3	5
Aspartic Acid 3TBDMS (418)	418.2 – 425.2	15	C18H40NO4Si3	5
Glutamic Acid 3TBDMS (432)	432.3 – 440.3	10	C19H42NO4Si3	6
Glutamine 3TBDMS (431)	431.3 – 439.3	10	C19H43N2O3Si3	6
Citric Acid 4TBDMS (591)	591.3 – 600.3	10	C26H55O7Si4	7
3-Phosphoglyceric Acid 4TBDMS (585)	585.4 – 592.4	15	C27H63O7PSi4	4

Supplementary Table 3: Realtime RT-qPCR primers used in this study for selcted human and murine genes of interest.

Gene Name	Species	Sequence Fwd (5' – 3')	Sequence Rev (5' – 3')
CDH1	human	AGTGCCAACTGGACCATTCA	TCTTTGACCACCGCTCTCCT
CycloA	human	CAGACAAGGTCCCAAAGACA	CCATTATGGCGTGTGAAGTC
GAPDH	human	CATGAGAAGTATGACAACAGCCT	AGTCCTTCCACGATACCAAAGT
MMP9	human	CGCGCTGGGCTTAGATCATT	GGTTCAGGGCGAGGACCATA
SLUG	human	CTACAGCGAACTGGACACACA	TGGAATGGAGCAGCGGTAGT
SNAIL	human	CGAAAGGCCTTCAACTGCAAA	TGACATCTGAGTGGGTCTGGA
VIM	human	CCTTGAACGCAAAGTGGAATC	GACATGCTGTTCCTGAATCTGAG
ZEB1	human	AAGAACTGCTGGGAGGATGACA	CCTCTTCAGGTGCCTCAGGAAAA
ZEB2	human	GCAAGAGGCGCAAACAAGC	GGGTTGGCAATACCGTCATCC
HMOX1	human	CTGCTCAACATCCAGCTCTTTG	ATCTTGCACTTTGTTGCTGGC
NFE2L2	human	AACTACTCCCAGGTTGCCCA	CAAGTGACTGAAACGTAGCCG
BACH1	human	GCCTCGCACAATATGGTTGAT	TGACATCGCACAGCACATCT
DHFR	human	AATCGGCTCAAAACCGCTTG	TCTGAATTCATTCCTGAGCGG
FolRa	human	TGGAACTGGACTTCAGGGTT	CACTCCCTCGGCTGTAGTTG
Zeb1	mouse	GGGAAATAGCACAGACAGTTGTT	TCACCTCTCCACAATGACGC
Zeb2	mouse	GTCGCTGTGTTTGGTTGCTAGA	CGTTGTCATAGTTCACCACGTT
Cdh1	mouse	TCATCAAATGGGGAAGCGGT	TTCATCACGGAGGTTCCTGG
Snail	mouse	GCTGCTTCGAGCCATAGAACTAAA	TTGAGGGAGGTAGGGAAGTGG
Vim	mouse	AGACCAGAGATGGACAGGTGA	CTGGTACTGCACTGTTGCAC
Gapdh	mouse	CATCACTGCCACCCAGAAGACTG	ATGCCAGTGAGCTTCCCGTTCAG
Sdha	mouse	CTTCGCTGGTGTGGATGTCA	GTGGGAATCCCACCCATGT
