# Supplementary material for: Mitochondria preserve an autarkic one-carbon cycle to confer growth-independent cancer cell migration and metastasis
Source: Nat Commun. 2022 May 16;13:2699. doi: 10.1038/s41467-022-30363-y (PMC9110368; doi:10.1038/s41467-022-30363-y)
Supplement: Supplementary file 2 — Reporting Summary [file 41467_2022_30363_MOESM2_ESM.pdf]

nature portfolio

Corresponding author(s): Johannes Meiser

Last updated by author(s): Mar 18, 2022

Reporting Summary

Nature Portfolio wishes to improve the reproducibility of the work that we publish. This form provides structure for consistency and transparency in reporting. For further information on Nature Portfolio policies, see our Editorial Policies and the Editorial Policy Checklist.

Statistics

For all statistical analyses, confirm that the following items are present in the figure legend, table legend, main text, or Methods section.

n/a	Confirmed	
☐	☒	The exact sample size (*n*) for each experimental group/condition, given as a discrete number and unit of measurement
☐	☒	A statement on whether measurements were taken from distinct samples or whether the same sample was measured repeatedly
☐	☒	The statistical test(s) used AND whether they are one- or two-sided<br>*Only common tests should be described solely by name; describe more complex techniques in the Methods section.*
☒	☐	A description of all covariates tested
☒	☐	A description of any assumptions or corrections, such as tests of normality and adjustment for multiple comparisons
☐	☒	A full description of the statistical parameters including central tendency (e.g. means) or other basic estimates (e.g. regression coefficient) AND variation (e.g. standard deviation) or associated estimates of uncertainty (e.g. confidence intervals)
☐	☒	For null hypothesis testing, the test statistic (e.g. *F*, *t*, *r*) with confidence intervals, effect sizes, degrees of freedom and *P* value noted<br>*Give P values as exact values whenever suitable.*
☒	☐	For Bayesian analysis, information on the choice of priors and Markov chain Monte Carlo settings
☒	☐	For hierarchical and complex designs, identification of the appropriate level for tests and full reporting of outcomes
☒	☐	Estimates of effect sizes (e.g. Cohen's *d*, Pearson's *r*), indicating how they were calculated

Our web collection on statistics for biologists contains articles on many of the points above.

Software and code

Policy information about availability of computer code

Data collection	GC-MS chromatograms were processed using Agilent MassHunter Quantitative Analysis for GC-MS, Version B.08.00<br>LC-MS Dara were acquired with Thermo Xcalibur software (Version 4.3.73.11)<br>WB: Detection was performed with the Odyssey CLx Infrared Imaging System using ImageStudioLite Software Vers.5.2<br>XF96 Extracellular Flux Analyzer (Seahorse Bioscience) was used to measure basal OCR following manufacturer's instructions using WAVE software Version 2.6.1.53.<br>Flow cytometric analysis was performed using BD FACSCanto (software BD FACSDiva 8.0.1), BD LSRFortessaTM (software BD FACSDiva 8.0.1), NovoCyte Quanteon (software NovoExpress 1.5.0)<br>Proliferation and Migration: IncuCyte Live-Cell Analysis system (Essen Bioscience)<br>QuantStudio 5 Real-Time PCR System (applied Biosciences, ThermoFisher Scientific) and data processing was performed using QuantStudio Design&Analysis Software v1.5.1 (Applied Biosciences, ThermoFisher Scientific)<br>For quantification of metastatic lung area images were acquired using BioTek Cytation 5 Cell Imaging Multimode Reader. Representative images for publication were acquired using Olympus IX83 microscope.
Data analysis	MetaboliteDetector software package (Version 3.220180913) was used for GC-MS mass spectrometric data post processing, quantification, MID calculations, correction of natural isotope abundance, and determinations of fractional carbon contributions<br>TraceFinder (Version 4.1) was used for LC-MS data processing. Subsequent data analysis for normalization and natural isotope subtraction were performed using in house scripts as in [Meiser, J., et al., Serine one-carbon catabolism with formate overflow. Sci Adv, 2016. 2(10): p. e1601273.]<br>ImageStudioLite Software Vers.5.2 (LI-COR) was used for image analysis and signal quantification of Western Blot<br>Analysis of flow cytometric data was performed in FlowJo Version 10.6.2<br>GraphPad Software Vers.8<br>For proteomics data, all data was analyzed with MaxQuant (version 1.6.7.0).

Wave Software Version 2.6.1.53 was used for Seahorse analysis.
IncuCyte ZOOM software 2018A, 2020B and 2021C were used to analyze IncuCyte Live-Cell Analysis.
RT qPCR data processing was performed using QuantStudio Design&Analysis Software v1.5.1 (Apllied Biosciences, ThermoFisher Scientific).
Quantification of lung area and percentage of H&E staining was performed in ImageJ.

For manuscripts utilizing custom algorithms or software that are central to the research but not yet described in published literature, software must be made available to editors and reviewers. We strongly encourage code deposition in a community repository (e.g. GitHub). See the Nature Portfolio guidelines for submitting code & software for further information.

Data

Policy information about availability of data

All manuscripts must include a data availability statement. This statement should provide the following information, where applicable:

- Accession codes, unique identifiers, or web links for publicly available datasets
- A description of any restrictions on data availability
- For clinical datasets or third party data, please ensure that the statement adheres to our policy

Source data are provided with this paper. Proteomics data are deposited on a server with the following pride ID: PXD027175. Proteomics data were analyzed and processed with Andromeda against the homo sapiens database from Uniprot, ProTIGY (https://github.com/broadinstitute/protigy), an R based tool, was used for differential analysis of MaxQuant output. At the current moment the access is set to private. It will be made public after acceptance of the manuscript. Access credentials for reviewers are as follows:
Username: reviewer_pxd027175@ebi.ac.uk; Password: 5Mhn9v58

Field-specific reporting

Please select the one below that is the best fit for your research. If you are not sure, read the appropriate sections before making your selection.

☒ Life sciences ☐ Behavioural & social sciences ☐ Ecological, evolutionary & environmental sciences

For a reference copy of the document with all sections, see nature.com/documents/nr-reporting-summary-flat.pdf

Life sciences study design

All studies must disclose on these points even when the disclosure is negative.

Sample size	For in vitro experiments, no statistical tests were applied to predetermine sample size. All experiments were performed in triplicate wells per condition (standard in the field). To control for experimental and biological variability each experiment was verified in independent experiments. The mean value of each individual experiment was used as one data point (one data point = 1 n) to calculate statistical significance. Specific number of repetitions is provided within the manuscript (figure legends) For in vivo work, number of mice per group was calculated based in a power calculation in liaison with a bio-statistician. Please see Material and Method section for further details.
Data exclusions	No data were excluded from the manuscript. The excluded samples in Figure 7 were excluded for technical reasons. the specific samples are shown in Figure S7D and explanation is provided in figure legend.
Replication	All in vitro experiments were verified in several independent experiments with the same overall outcome. Specific number of repetitions is provided within the manuscript (figure legends). For ethical reasons, the in vivo work was only done once. However, adequate group size to obtain sufficient statistical power was calculated beforehand (please see Material and Method section).
Randomization	For in vitro cell culture experiments cells were randomly seeded from one cell population. Plates and lanes were randomly allocated to conditions.<br>Before start of the in vivo experiment, mice were randomly allocated to the different groups.<br>All Mass Spec samples were randomized during the analysis to avoid bias from e.g. instrument drift.
Blinding	Group allocation of mice was randomized. Investigators were blinded during sample collection of in vivo experiment (only IDs were provided). Analyses of in vivo samples were blinded and analyzed by two persons independently. IDs were uncovered after final result was obtained. Results from both persons (macrometastasis counting) lead to the same overall result/interpretation.

Reporting for specific materials, systems and methods

We require information from authors about some types of materials, experimental systems and methods used in many studies. Here, indicate whether each material, system or method listed is relevant to your study. If you are not sure if a list item applies to your research, read the appropriate section before selecting a response.

Materials & experimental systems

n/a	Involved in the study	
	x	Antibodies
	x	Eukaryotic cell lines
x		Palaeontology and archaeology
	x	Animals and other organisms
x		Human research participants
x		Clinical data
x		Dual use research of concern

Methods

n/a	Involved in the study	
x		ChIP-seq
	x	Flow cytometry
x		MRI-based neuroimaging

Antibodies

Antibodies used	MTHFD1L (16113-1-AP, LOT: 00024322, RRID: AB_2250974) from Proteintech; Vimentin (3390, clone 5G3F10, LOT:2, RRID: AB_2216128), β-actin (3700, clone 8H10D10, LOT: 18, RRID: AB_2242334), NRF2 XP(R) (12721, clone D179C, LOT: 8, RRID: AB_2715528) and MTHFD2 (41377, D8W9U, LOT: 1, RRID: AB_2799200) from Cell Signaling Technology (CST); PHGDH (HPA021241, LOT: B115626, RRID: AB_1855299), PSAT1 (HPA042924, LOT: R40839, RRID: AB_2678223), PSPH (HPA020376, LOT: B115600, RRID: AB_1855867), SHMT1 (HPA023314, LOT: A78402, RRID: AB_1856830), and SHMT2 (HPA020549, LOT: A96534, RRID: AB_1856834) from Sigma Aldrich; LaminB (ab16048, LOT: GR32836742, RRID: AB_443298) from Abcam; IRDye 680RD Goat Anti-Mouse IgG (H+L) (926-68070, LOT: C90910-21, RRID: RRID AB_10956588) and IRDye 800CW Donkey Anti-Rabbit IgG (H+L) (926-32213, LOT: 926-32213, RRID: RRID AB_621848) from LI-COR (used in a 1:10,000 dilution)<br>All antibodies were used in a 1:1,000 dilution unless stated otherwise.
Validation	All antibodies used in this study are commercially available and have been declared to be tested by the manufacturer for species and target reactivity. All related information can be found on the supplier's webpage in the respective datasheet or certificate of analysis. Clones, catalogue and RRID numbers for each antibody have been included (please see "Antibodies used") for referring to the manufacturing company specification/validation processes. We further validated antibodies in house by using negative as well as positive controls where possible (using cell populations known to lack or express a certain marker).

Eukaryotic cell lines

Policy information about cell lines

Cell line source(s)	MDA-MB-468: Weill Cornell Medical College, USA, Lewis Cantley lab (authenticated by our lab in 2021) (originally from ATCC); LN229: Beatson Institute for Cancer Research, Alexei Vazquez lab (authenticated by our lab in 2021) (originally from ATCC);HAP1: MRC laboratory, Cambridge UK, KJ Patel lab; 4T1: Luxembourg Institute of Health, Clement Thomas lab (authenticated by Clement lab in 2018) (originally from Horizon Discovery).<br>HCT-116: University of Luxembourg, Elisabeth letellier lab (originally from ATCC), HEK293T: NorLux Lab, Luxembourg Institute of Health (originally from ATCC)
Authentication	MDA-MB-468, 4T1 and LN229 cells were authenticated by STR Profiling service of Eurofins Genomics Europe Genomics GmbH and subsequent comparative analysis using CLASTR 1.4.4 under https://web.expasy.org/cellosaurus-str-search/. HEK293T, HAP1 and HCT116 cells were not authenticated.
Mycoplasma contamination	Cells were monthly checked for mycoplasma contamination and were always mycoplasma free.
Commonly misidentified lines<br>(See ICLAC register)	No commonly misidentified cell lines (listed in the ICLAC database, version 11) were used in this study.

Animals and other organisms

Policy information about studies involving animals; ARRIVE guidelines recommended for reporting animal research

Laboratory animals	8-10 week old female Balb/c mice (9 animals per group) were used for the in vivo work.
Wild animals	No wild animals were used in this study.
Field-collected samples	No field collected samples were used in this study.
Ethics oversight	Animal experiments were performed according to all applicable laws and regulations, after receiving approval by the institution's Animal Experimentation Ethics Committee at UL (AEEC) and the Luxembourgish veterinarian service of the Ministry of Agriculture, Viniculture & Rural Development (TumorMetab LUPA 2020/01). They ensure that care and use of animals for research purposes was conducted according to the EU Directive 2010/63/EU, as well as the Grand-Ducal Regulation of January 11, 2013 on the protection of animals used for scientific purposes.

Note that full information on the approval of the study protocol must also be provided in the manuscript.

Flow Cytometry

Plots

Confirm that:

- [x] The axis labels state the marker and fluorochrome used (e.g. CD4-FITC).
- [x] The axis scales are clearly visible. Include numbers along axes only for bottom left plot of group (a 'group' is an analysis of identical markers).
- [x] All plots are contour plots with outliers or pseudocolor plots.
- [x] A numerical value for number of cells or percentage (with statistics) is provided.

Methodology

Sample preparation	200,000 cells were seeded in 2 ml DMEM and treated the subsequent day as indicated. After incubation, medium was collected and cells were washed with PBS. PBS fraction was collected and cells were detached with trypsin. Detached cells were collected in DMEM. The combined, collected solutions were centrifuged and pellet was washed with PBS.<br>Cell Cycle Distribution<br>Centrifugation yielded a pellet that was resuspended in 100 µl PBS and fixed with ice-cold 80% EtOH. Fixed cells were stored at -20°C for at least 1 h and maximum 5 days prior to measurement. Cells were centrifuged, pellet was incubated for 1 h in 200 µl RNAse A in PBS (30 µg/ml) at RT. Immediately prior to measurement, 98 µl propidium iodide (PI) in PBS (50 µg/ml) was added. Flow cytometric analysis was performed using BD FACSCanto and BD FACSDiva software. Analysis was performed in FlowJo Version 10.6.2.<br>Cell Death Analysis<br>Pellet following centrifugation was resuspended in 50 µl AnnexinV-FITC staining solution (5% AnnexinV-FITC in AnnexinV binding buffer (10 mM HEPES pH 7.4, 140 mM NaCl, 2.5 mM CaCl2, 0.1% BSA in ddH2O)) and incubated for 15 min on ice in the dark. 450 µl PI-staining solution (1.1 µg/ml PI in AnnexinV binding buffer) was added immediately prior to measurement using BD FACSCanto and BD FACSDiva software (for wild-type cells) or NovoCyte Quanteon (for GFP-positive transfected cells). Analysis was performed in FlowJo Version 10.6.2.<br>Flow Cytometric Analysis of ROS Levels<br>250,000 MDA-MB-468 cells were seeded in 2 ml medium and treated the subsequent day as indicated. Following incubation, cells were detached with trypsin, centrifuged at 350 g for 5 min and washed with warm DMEM. Cells were stained in 100 µl DMEM supplemented with 1:2,000 DAPI and 1:500 DCFDA for 30 min at 37°C. Following incubation, samples were centrifuged at 350 g for 5 min and washed with PBS. Following centrifugation at 350 g for 5 min, cells were resuspended in 100 µl PBS and measured using the BD LSRFortessaTM system and BD FACSDiva software. Data analysis was performed using FlowJo software Version 10.6.2.
Instrument	BD FACSCanto (338073), NovoCyte Quanteon (4025), BD LSRFortessaTM system
Software	BD FACSDiva software; FlowJo Software (Vers 10.6.2)
Cell population abundance	All experiments were performed with a pure cancer cell line. Flow cytometry was not used to enrich certain populations as the cell lines are already 100% pure.
Gating strategy	FSC/SSC selection of cells, exclusion of doublets by FCS-A/FSC-H<br>Further gating strategies done according to established protocols and in dependence of the individual staining (see Material and Methods section). No special gating strategy to be defined (no special gating done; only exclusion of cell debris, aggregates and doublets)

- [x] Tick this box to confirm that a figure exemplifying the gating strategy is provided in the Supplementary Information.
